# Effects of Postoperative Rehabilitation on Gait Parameters and Electromyography Variables in Acute and Chronic Anterior Cruciate Ligament Reconstruction Surgery in Football Players

**DOI:** 10.1155/2021/9912795

**Published:** 2021-08-13

**Authors:** Gopal Nambi, Walid Kamal Abdelbasset, Anju Verma, Shereen H. Elsayed, Osama R. Aldhafian, Naif Bin Nwihadh, Mohamed A. Omar, Tohamy G. T. Hassan, Ayman K. Saleh

**Affiliations:** ^1^Department of Health and Rehabilitation Sciences, College of Applied Medical Sciences, Prince Sattam Bin Abdulaziz University, Al-Kharj, Saudi Arabia; ^2^Department of Physical Therapy, Kasr Al-Aini Hospital, Cairo University, Giza, Egypt; ^3^Department of Rehabilitation Sciences, Faculty of Health and Rehabilitation Sciences, Princess Nourah Bint Abdulrahman University, Riyadh, Saudi Arabia; ^4^Department of Cardiovascular/Respiratory Disorder and Geriatrics, Faculty of Physical Therapy, Cairo University, Giza, Egypt; ^5^Department of Surgery, College of Medicine, Prince Sattam Bin Abdulaziz University, Al-Kharj, Saudi Arabia; ^6^Department of Orthopedic Surgery, Faculty of Medicine for Girls, Al-Azhar University, Cairo, Egypt

## Abstract

**Results:**

The results of the a-ACLR, c-ACLR, and control groups were compared. At 8 weeks following postoperative rehabilitation, the a-ACLR group shows more significant changes than the c-ACLR group (*p* < 0.001). At 6 and 12 months, there are normal values of kinematic and kinetic values in a-ACLR compared with the results of the control group (*p* < 0.001).

**Conclusion:**

The study showed that postoperative rehabilitation provides significant effects in the kinematic, kinetic, and EMG gait parameters in acute ACLR than chronic ACLR subjects. Early surgical intervention and postrehabilitation are mandatory to get the significant effects in the clinical parameters in acute and chronic ACL injury.

## 1. Introduction

Anterior cruciate ligament (ACL) injury commonly occurs in the game of football and it is categorized into acute (a-ACL) (<1 month) and chronic (c-ACL) (>2 years) injury [1]. Biomechanically, a-ACL injury subjects exhibit a quadriceps avoidance gait which is seldom reported in c-ACL injury subjects [2]. This specific “quadriceps avoidance gait” in an ACL injured subject is a peculiar type of gait, which is occurred due to pain, edema, abnormal motion, muscle wasting, muscle adaptation, or neuromuscular reprogramming [3]. These changes in gait can be observed through a motion analysis system, which is an advanced device to dynamically evaluate the joint movements, providing precise information about ACL deficient knees [4].

Clinically, both types of ACL injuries are reported with decreased knee flexion in gait. Clinical observatory changes are noted in the initial foot contact phase (0% to 10%) and midstance phase (10% to 30%) of the gait cycle, which lead to antero-posterior instability in the knee joint [5]. This pattern of gait reduces the hamstring muscle activity during the midstance phase, which increases the forward translation force of the tibia. Andriacchi and Dyrby observed that this anterior translation of the tibia is done by the strong eccentric action of quadriceps muscles at initial knee flexion. It is also found that there is a muscular imbalance between the flexors and extensors of the knee and reduced knee flexor function [6]. Studies also reported the biomechanical compensations in ACL injured subjects at the terminal stance phase (30% to 50%), which shows a substantial amount of stress on ACL during the final degree of knee extension. The ACL injured knee also loses its screw-home mechanism, which causes unstable knee joints [7].

Anterior cruciate ligament reconstruction (ACLR) surgery is a widely used surgical intervention in the management of acute and chronic ACL injuries. In this surgery, the patellar tendon or quadrupled semitendinosus/gracilis tendon has been used to replace the ACL rupture [8]. Surgical reconstruction of the ACL could lead to changes in the gait parameters, force production, and lower limb muscle activities [9]. Different ACL reconstruction rehabilitation protocols were primarily framed based on the type of surgery and its involvement. The benefit of ACLR rehabilitation guidelines is that they maximize the speed of a patient's progress through the use of subjective and objective measures of impairment and level of function. These postoperative ACLR rehabilitation protocols are helpful to minimize the pain and effusion, improve the knee range of motion (ROM) and knee muscles strength, and normalize the gait pattern. Appropriate decision-making by the rehabilitation team based upon predetermined criteria in these protocols is required to optimize outcomes and allow for a safe return to sport [10].

Few trials have individually evaluated the changes in gait parameters following acute ACLR (a-ACLR, surgery performed within one month of injury) and chronic ACLR (c-ACLR, surgery performed greater than two years of injury) surgeries. Devita et al. [11] compared the gait parameters of a-ACLR surgery and found that different time durations such as 2 weeks postinjury and 5 weeks after surgery elicited quadriceps avoidance gait. Bush-Joseph et al. [12] conducted a study on a-ACLR participants after 8 months postsurgical follow-up and found minimal clinical changes in flexion knee range and quadriceps avoidance gait pattern during walking. Butler et al. [13] analyzed the gait parameters of c-ACLR participants and found no such substantial biomechanical changes in gait pattern and muscle activities. Comparing these results to those of Moraiti et al., [14] c-ACLR subjects with 8 weeks of postrehabilitation show better results in gait patterns and muscle electromyography (EMG) activities than a control group. However, few studies have compared the gait patterns after postoperative rehabilitation following acute and chronic ACL reconstruction surgeries [14, 15].

To date, there was no clinically proven and evident ACLR rehabilitation protocol developed specifically for football players in the sports rehabilitation field, and also no comparative studies were conducted on kinematic, kinetic, and EMG parameters after 8 weeks of rehabilitation protocol in acute and chronic ACLR subjects. The execution of this study would be clinically helpful for developing a sports-specific rehabilitation protocol for the ACLR subjects and also evidence for the ACL injured subjects to go for the early surgical correction and rehabilitation. Hence, the objective of this trial was to investigate the kinematic, kinetic, and EMG effects of postoperative rehabilitation after acute and chronic ACLR surgery in football players. The null hypothesis of this study is that there are no differences in kinematic, kinetic, and EMG parameters in postoperative rehabilitation after acute and chronic ACLR surgery in football players.

## 2. Materials and Method

### 2.1. Trial Design

The study design was a quasiexperimental study, and the subjects were selected using convenience sampling. Totally, forty-five subjects participated in this study; a-ACLR (*n* = 15) and c-ACLR (*n* = 15) subjects were compared with (*n* = 15) healthy football players. Basic examinations and special tests were performed on all the subjects by an orthopedic surgeon for consideration to be included in the study and these data were not considered for statistical analysis.

### 2.2. Participants

#### 2.2.1. Eligibility Criteria

In order to participate in the study, the subject should be a university football player, who has undergone either acute or chronic ACLR surgery in one leg with less than 2 mm antero-posterior translation, normal contralateral limb, not to have any other pathologies and soft tissue injuries in the affected extremity, absence of “giving way,” no use of analgesic drugs following surgery, no postoperative consequences, and who agreed to be in the trial. For inclusion, in the reconstruction surgery, the subjects should have undergone only conservative treatment without any other soft tissue injuries such as menisci, medial collateral ligament, and lateral collateral ligament. For inclusion in the control group, the subjects should be healthy and willing to participate voluntarily. The participants with muscle and joint injuries, subluxation, dislocation, fracture at the femur and tibia, previous surgeries, and neurological and orthopedic problems of the lower extremities were excluded from the study. This trial was conducted in the department of physical therapy, Prince Sattam Bin Abdulaziz University, Al-Kharj, Saudi Arabia.

### 2.3. Interventions

The a-ACLR group consists of 15 acute ACL injured subjects (less than 1 month) in the range of (8 to 24 days) and the c-ACLR group consists of 15 chronic ACL injured subjects (more than 24 months–2 years) in the range of (28 to 46 months). The control group consists of 15 healthy football players who were examined and allocated in the study. Participants in the three groups were asked to read and sign an informed participant form which was agreed upon by the institutional departmental ethical committee. The postoperative ACLR rehabilitation protocol (Annexure-1) was also agreed by the institutional departmental ethical committee (RHPT/018/23). This trial was executed as per the strict guidelines of the Declaration of Helsinki 1964.

**Figure 1:**
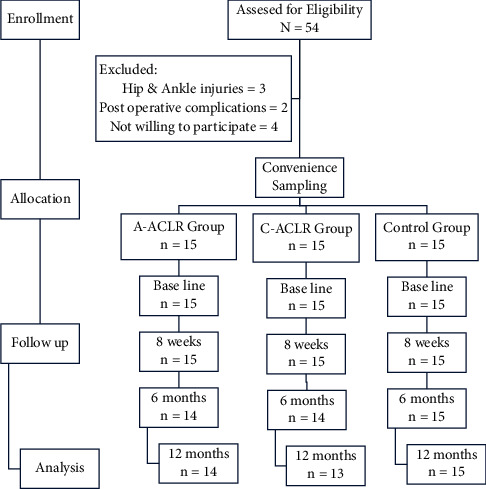
Flow chart for distribution of subjects and follow-up analysis.

The 8 weeks postoperative ACLR rehabilitation protocol was executed by a trained physical therapist, as per the directions from an orthopedic surgeon. The rehabilitation protocol specifically laid stress on the strengthening of quadriceps and hamstring muscles. The subjects were instructed about dos and don'ts at home by the treating therapist through a pamphlet. [16] The study excluded three subjects with other joint injuries (the hip and ankle), two with postoperative complications (pain and swelling) and four who did not agree to sign the informed participant form (Figure 1).

### 2.4. Instrumentation

A VICON motion analysis system (VICON MX +  motion capture system, Oxford, UK) was used to analyze the kinematic data of gait. Twelve-infrared light MX+ and 2-megapixel camcorders (frequency of 200 Hz) were fixed in various directions to measure the kinematic movements of the pelvis, hip, knee, and ankle joints. Four parts of the lower extremity such as the pelvis, thigh, leg, and foot 3D movements were recorded through reflective markers [17]. The disc-shaped retro-reflective markers (25 mm) were placed on different parts of the limb to measure the kinematic data. This method of motion analysis with a motion analysis system has good validity and reliability for measuring three-dimensional kinematic analysis of the gait [18].

Kinetic data were measured through force plates (ADAL3DM-F-COP-Mz, Medical Development, France), which were implanted in the floor. It was used to measure the three-dimensional (anterior-posterior, medial-lateral, and vertical) force reactions and point of pressure variance at each limb. The whole motion analysis system and the force plates were calibrated before the initiation of the study.

Electromyography (EMG) data were measured for (VM, vastus medialis) (VL, vastus lateralis) (BF, biceps femoris) (AL, adductor longus) muscles of both sides because these are the important dynamic stabilizers of the knee. The EMG signals were recorded through small plate electrodes (BlueSensor P-00-S, Germany) and precisely positioned in the proper place (muscle belly) to avoid the crosstalk. The skin was cleaned with soap water and rubbed with an alcohol solution to reduce the skin resistance and to obtain an optimal signal. The subject performed submaximal voluntary contraction (sMVC) to isolate the individual muscle groups in the nonoperated leg. The obtained analog EMG signal was preamplified with high-range filters for producing digital signals. An average of six gait cycles has been calculated and the final EMG curve was normalized and plotted [19].

### 2.5. Dependent Variables

For measuring kinematic and kinetic analysis, the subject was relaxed and allowed to walk six times identically in the gait mat with a self-selected, fixed comfortable speed of 1 m/s for a distance of 8–10 m without shoes. Among the recorded walks, one best walk (according to the fixed speed 1 m/s) was selected and sent forward for processing to analyze the spatiotemporal gait parameters. Five kinematic parameters were considered in the study as follows: (1) cadence (no. of steps/minute), (2) step length (cm), (3) step width (cm), (4) double support (% of the gait cycle), and (5) swing phase (% of the gait cycle). The kinetic ground reaction forces such as maximum force at (1) F1, early stance phase; (2) F2, middle stance phase; and (3) F3, late stance phase forces, were also recorded. The data collected through the camcorders were processed using Nexus software and converted into digital form. [20].

The EMG activities of specific muscles were measured in hundred points (1% to 100%) gait cycle. Group means muscle activities were compared during various phases of gait between a-ACLR, c-ACLR, and control groups. The outcome variables were measured four times: immediately before surgery (first measurement), 8th week (second measurement), 6th month (third measurement), and 12th month (fourth measurement) after ACLR.

### 2.6. Sample Size

The total number of subjects required for this study was 45 which was obtained by G^*∗*^ power statistics. It was assumed with a statistical power of 80% with identifying minimum clinical difference score of 40% in VAS pain intensity with a standard error of 2 and the significant level was set at *α* = 0.05.

### 2.7. Blinding

The researcher who did not measure the outcome variables performed the group allocation. The eligible subjects in each group were selected through a convenience sampling method. Subjects in all three groups received postoperative ACLR rehabilitation protocol according to their group assignment. Due to this study design, it was not possible to do blinding of the therapist and the subjects. The researcher who was assessing the outcome measures at various durations was blinded.

### 2.8. Statistical Analysis

Subject baseline characters such as age, height, weight, BMI, heart rate, duration of injury, and years of playing were noted, and the study homogeneity was calculated using the “Kruskal–Wallis” test. All the outcome variables were calculated as class intervals for 95% with mean and standard deviation. Repeated measure analysis of variance (ANOVA) was done to calculate significant differences in gait scores within groups. One-way analysis of variance (ANOVA) was done to compare the gait scores between the groups and the statistical significance level was set at *α* = 0.05. SPSS software (version 19.0), SPSS Inc., Chicago, Illinois, USA, was used for doing all statistical tests.

## 3. Results

The subject's baseline characters such as age, height, weight, BMI, heart rate, and years of playing did not find any difference between the three groups (Table 1).

### 3.1. Kinematic Variables

Table 2 shows the comparative summary of kinematic parameters of a-ACLR, c-ACLR, and control subjects at baseline, 8 weeks, 6 months, and 12 months. The intragroup analysis through repeated measure ANOVA shows considerable statistical differences (*p* < 0.05) in cadence (steps/min), step length (cm), and swing phase (% of the gait cycle) of the a-ACLR and c-ACLR groups, but not in step width and double support (*p* > 0.05).

Intergroup (a-ACLR, c-ACLR, and control groups) analysis through one-way ANOVA in cadence, step length, and swing phase shows significant changes (*p* < 0.001). But at the same time, step width and double support did not show any statistical difference (*p* > 0.05) between the three groups. The scores of cadence, step length, and swing phase of a-ACLR and c-ACLR groups are becoming closer to the scores of the control group at 6 and 12 months. In all kinematic gait parameters, the a-ACLR (MCID: cadence, 45.1; step length, 40.2; step width, 5.6; double support, 4.39; swing phase, 10) subjects show marked response compared to the c-ACLR (MCID: cadence, 24.0; step length, 49.9; step width, 3.4; double support, 4.82; swing phase, 5.79) and control group and the c-ACLR and control group scores show similar values.

### 3.2. Kinetic Variables

The force is described by the relative force applied by the foot to the ground during the stance phase. Three characterized points such as F1, early stance phase; F2, midstance phase; and F3, late stance phase, are considered for analysis. The analysis of this parameter shows significant increase (*p* < 0.001) values in a-ACLR (MCID: F1, early stance phase, 15.0; F2, middle stance phase, 15.0; F3, late stance phase, 15.0) compared to c-ACLR (MCID: F1, early stance phase, 10.0; F2, middle stance phase, 9.0; F3, late stance phase, 9.0) and control subjects. No marked changes were noted between the c-ACLR and healthy control group subjects following 6 and 12 months of surgery (Table 3).

### 3.3. Electromyographic Variables

**Figure 2:**
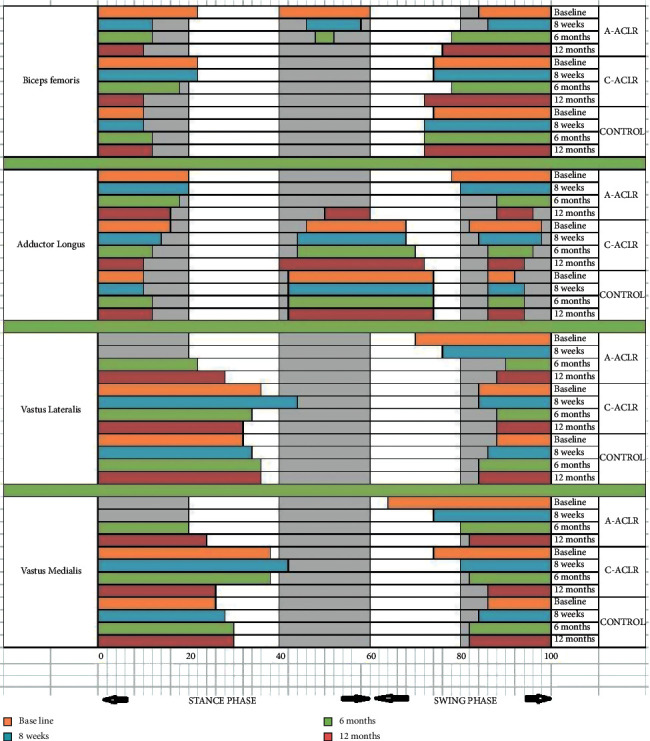
Electromyography (EMG) analysis of biceps femoris, adductor longus, vastus lateralis, and vastus medialis muscles in a-ACLR, c-ACLR, and healthy control groups at baseline, 8 weeks, 6 months, and 12 months.

Immediately after surgery, the biceps femoris muscle elicited marked EMG activity in the midstance phase (40%–60%) in the a-ACLR group compared to c-ACLR and control groups. Following 8 weeks of postrehabilitation and at 12 months follow-up period, the marked activity is reduced in the a-ACLR group compared to c-ACLR and control groups. At baseline, the adductor longus muscle shows an absence of EMG activity in midstance phase (40%–60%) in a-ACLR subjects. Later at 12 months follow-up period, there is a slight improvement in the activity of adductor longus muscle in the a-ACLR group and marked improvement in the c-ACLR group. In early stance phase (0%–20%), there is an absence of vastus lateralis and vastus medialis EMG activity. Later at 6 months and 12 months follow-up, these muscle activities are increased in a-ACLR subjects. In the c-ACLR group, the vastus lateralis and vastus medialis muscles are activated more during the baseline and 8 weeks evaluation and gradually reduced after 12 months follow-up; it shows a longer time to recover and at 6 and 12 months shows considerable changes compared to the control group (Figure 2).

## 4. Discussion

The gait which a-ACLR subjects used before surgery is “quadriceps avoidance gait” and c-ACLR subjects used is “unstable gait.” The overall results of this study show relatively improved gait parameters in both groups when compared to a healthy control group [21]. Biomechanically, the final stance phase of gait kept a significant strain on the ACL due to internal tibial force which shows the relation between knee joint stability and flexion range of motion [22]. In the case of ACL injury, when the subject stands on the affected leg during midstance, the quadriceps pulls the tibia anteriorly and the femur shifts posteriorly which leads to anterolateral rotatory knee instability [23].

In the a-ACLR group, there is clear evidence of quadriceps avoidance pattern before surgery, due to an action of increased extension at the knee in stance phase and decreased flexion at the knee in the swing phase [2]. This pattern is maintained by the reduction of knee extensor (vastus medialis and vastus lateralis) and adductor longus activity and an increase in biceps femoris (synergist) function. This muscle coordination maintains the knee joint stability during the stance phase by minimizing internal rotation (pivoting), which was reviewed and accepted [24]. In addition, the relative motion of the tibia over the femur is increased and also noted that the biceps femoris muscle acts as a powerful supporter to increase the knee joint stability [2, 25].

In the c-ACLR group, there is evidence of unstable gait pattern because the kinematic analysis shows changes in gait pattern and shows the significant difference with the control group. Studies report that 57% to 80% of chronic ACL injured subjects exhibit quadriceps avoidance patterns. The knee extensor muscle elicits longer muscle activity, which is supported and contrasted by some studies [25, 26]. On and above, c-ACLR subjects show reduced tibial translation when compared to the a-ACLR group, but a significant difference was observed between c-ACLR and control groups during the examination. This is due to the long activity of the knee extensor, which is observed in the Chmielewski et al.'s study [22]. The study also depicted high hamstrings activation (biceps femoris) which is known as hamstring facilitation acts to control the tibial translation during the preswing phase [22, 25, 26] which was proved by EMG studies.

During the baseline analysis immediately after a-ACLR and c-ACLR, we observed significantly reduced spatiotemporal kinematic parameters compared with the control group at stance and swing phase. After 8 weeks of rehabilitation, the kinematic gait parameters were improved at 6 and 12 months in the a-ACLR group than the c-ACLR group. However, more time is required to obtain the normal tibial translation and the report was supported by Claes et al. [27] and contradicted by Chen et al. [20].

Implementation of the 8 weeks rehabilitation protocol may also affect the kinetic gait parameters [28, 29]. We observed reduced force creation (F1, F2, and F3) in the acute or chronic ACL injured limbs when compared to the control group. At the end of 8 weeks postrehabilitation, the force parameters were restored considerably. Significant improvement in force production was observed in the a-ACLR group than the c-ACLR group at 6 and 12 months [30–32].

Eight weeks of postoperative rehabilitation also showed changes in EMG muscle activities, which were observed in all three groups. The EMG analysis shows that at least 6 months are required for the a-ACLR subjects and more than 6 months are required for c-ACLR subjects to become normal. Our reports are in agreement with Chmielowski et al. and found that the quadriceps muscle activity is diminished in both acute and chronic ACL reconstructed patients during the stance phase of gait. Also, he observed that the adductor longus muscle and biceps femoris muscle activity is improved in the terminal stance phase after postoperative rehabilitation [22]. Same way to reestablish and rehabilitate the normal biomechanical activity of gait after ACLR takes time and is consistent with the results of Chmielewski et al. [22].

The greater strength of this study is its real-time measurements of kinematic, kinetic, and EMG analysis in acute and chronic ACLR subjects. Still, a few limitations have been noted and considered while executing this study. First, the sample population was small, and therefore, a generalization of the results becomes difficult. Secondly, the measures such as pain intensity, muscle strength, and functional status were not considered for the data interpretation. Finally, we have not found the intragroup difference between the 8 weeks of sports-specific rehabilitation group and conventional training in acute and chronic ACLR.

In conclusion, the study indicates that time of injury and intervention plays a major role in the rehabilitation of ACL injury, in adapting normal gait parameters and muscle activity. The study showed that sports-specific rehabilitation protocol provides significant effects in the kinematic, kinetic, and EMG gait parameters in acute ACLR than chronic ACLR subjects. Future studies should look forward to formatting fast rehabilitation protocols to reduce the clinical visit and reestablish the gait parameters quickly.

## Figures and Tables

**Table 1:** Demographic variables of a-ACLR, c-ACLR, and control groups.

Sr. No.	Variable	a-ACLR	c-ACLR	Control	*p* value
1	Age (y)	19.86 ± 4.52	19.72 ± 2.15	19.86 ± 4.32	0.996
2	Height (m)	1.77 ± 0.03	1.74 ± 0.05	1.75 ± 0.04	0.336
3	Weight (kg)	71.42 ± 6.06	78.13 ± 6.23	75.22 ± 5.26	0.085
4	BMI	22.8 ± 1.6	23.1 ± 1.7	23.2 ± 1.5	0.777
5	Heart rate	75.21 ± 3.21	76.11 ± 2.91	76.64 ± 3.15	0.447
7	Years of playing	3.22 ± 1.08	3.18 ± 1.22	3.82 ± 1.52	0.653

**Table 2:** Kinematic variables of a-ACLR, c-ACLR, and control groups.

Group	Baseline	8 weeks	6 months	12 months	*p* value
Cadence (steps/min)					
a-ACLR	40.2 ± 08.2	55.2 ± 09.2	72.1 ± 04.5	85.3 ± 08.2	0.001
c-ACLR	58.6 ± 07.5	65.3 ± 12.5	72.6 ± 10.2	82.6 ± 09.2	0.001
Control	85.6 ± 13.5	86.6 ± 13.5	89.3 ± 10.5	90.2 ± 11.2	0.768
*p* value	0.001	0.001	0.001	0.001	—

Step length (cm)					
a-ACLR	280.5 ± 12.4	310.5 ± 12.2	345.2 ± 10.8	320.7 ± 11.8	0.001
c-ACLR	310.5 ± 10.4	324.8 ± 08.2	342.2 ± 12.2	360.4 ± 13.4	0.001
Control	362.5 ± 12.5	363.5 ± 10.5	364.5 ± 12.5	366.5 ± 10.1	0.849
*p* value	0.001	0.001	0.001	0.001	—

Step width (cm)					
a-ACLR	27.8 ± 05.4	25.2 ± 03.4	23.0 ± 05.8	22.2 ± 06.2	0.254
c-ACLR	25.9 ± 03.2	23.9 ± 05.6	22.0 ± 03.6	22.5 ± 04.3	0.127
Control	23.2 ± 05.6	22.5 ± 08.6	22.3 ± 07.1	22.2 ± 05.6	0.984
*p* value	0.081	0.576	0.653	0.717	—

Double support (% of gait)					
a-ACLR	15.61 ± 4.8	14.28 ± 3.6	12.28 ± 3.2	11.22 ± 7.8	0.431
c-ACLR	16.39 ± 3.6	15.82 ± 4.8	12.52 ± 2.9	11.57 ± 3.4	0.521
Control	12.52 ± 2.9	12.32 ± 2.2	12.11 ± 0.8	11.32 ± 0.6	0.434
*p* value	0.082	0.081	0.525	0.899	—

Swing phase (% of gait)					
a-ACLR	54.22 ± 3.1	51.01 ± 2.7	46.36 ± 4.1	44.22 ± 3.5	0.001
c-ACLR	50.71 ± 2.8	48.22 ± 3.6	46.21 ± 3.1	44.92 ± 2.2	0.001
Control	44.21 ± 2.8	43.86 ± 1.9	43.01 ± 1.8	43.01 ± 2.5	0.474
*p* value	0.001	0.001	0.001	0.027	—

**Table 3:** Kinetic variables of a-ACLR, c-ACLR, and control groups.

Group	Baseline	8 weeks	6 months	12 months	*p* value
F1, early stance phase					
a-ACLR	128 ± 0.5	130 ± 0.3	142 ± 0.4	143 ± 0.6	0.001
c-ACLR	132 ± 0.4	136 ± 0.4	140 ± 0.4	142 ± 0.5	0.001
Control	142 ± 0.6	143 ± 0.5	143 ± 0.2	144 ± 0.6	0.001
*p* value	0.001	0.001	0.001	0.001	—

F2, middle stance phase					
a-ACLR	128 ± 0.2	131 ± 0.4	138 ± 0.3	143 ± 0.4	0.001
c-ACLR	133 ± 0.4	135 ± 0.4	140 ± 0.3	142 ± 0.3	0.001
Control	143 ± 0.5	143 ± 0.4	145 ± 0.3	145 ± 0.3	0.001
*p* value	0.001	0.001	0.001	0.001	—

F3, late stance phase					
a-ACLR	126 ± 0.3	128 ± 0.5	138 ± 0.2	141 ± 0.5	0.001
c-ACLR	131 ± 0.3	134 ± 0.4	138 ± 0.5	140 ± 0.4	0.001
Control	140 ± 0.8	141 ± 0.3	142 ± 0.3	142 ± 0.4	0.001
*p* value	0.001	0.001	0.001	0.001	—

## Data Availability

Data are available on request through accessing the corresponding author.

## Supplementary Materials

Supplementary Materials The postoperative ACLR rehabilitation protocol (Annexure-1).
