# Supplementary material for: Effects of Postoperative Rehabilitation on Gait Parameters and Electromyography Variables in Acute and Chronic Anterior Cruciate Ligament Reconstruction Surgery in Football Players
Source: Evid Based Complement Alternat Med. 2021 Aug 13;2021:9912795. doi: 10.1155/2021/9912795 (PMC8378949; doi:10.1155/2021/9912795)
Supplement: Supplementary Materials — The postoperative ACLR rehabilitation protocol (Annexure-1). [file 9912795.f1.docx]

**Annexure - 1**

| ACLR Post Surgical Rehabilitation Protocol - Football | |
| --- | --- |
| Phase – I Early Post-operative phase (0-2 weeks) | |
| Swelling Management: Ice, compression, elevation, Retrograde massage, Ankle pumps | |
| Range of motion/Mobility:  Patellar mobilizations: superior/inferior & medial/lateral.  Seated assisted knee flexion extension and heel slides with towel.  Low intensity, long duration extension stretches: prone hanging, heel prop.  Standing gastrocnemius and soleus stretch.  Supine active hamstring stretch and supine passive hamstring stretch. | **Precautions:**  Weight bearing: Weight bearing as tolerated with crutches.  Brace: Post-operative extension brace for 2-4 weeks.  Range of Motion: Goal of 0-90° within 1 week, moving toward full flexion after the first 4 weeks. |
| Strengthening: Calf raises / Quad sets, Hip abduction/side leg lift  Multi-angle isometrics 90 and 60 deg knee extension.  NMES: High intensity (2500 Hz, 75 bursts)  supine knee extended 10 sec/50 sec, 10 contractions, 2x/wk Straight leg raise | **Criteria to Progress:**  Knee extension ROM 0 deg.  Quad contraction with superior patella glide and full active extension.  Able to perform straight leg raise without lag. |
| Phase – II Intermediate Post-operative phase (3-6 weeks) | |
| Range of motion/Mobility:  Stationary bicycle.  Gentle stretching all muscle groups: Prone quad stretch, standing quad stretch, kneeling hip flexor stretch | **Precautions:**  Full weight bearing  Avoid over-loading the fixation site by utilizing low amplitude low velocity movements.  No active inflammation or reactive swelling. |
| Strengthening:  Prone hamstring curls, Step ups and step ups with march, Partial squat exercise  Ball squats, wall slides, mini squats from 0-60 deg.  Lumbo pelvic strengthening: bridge & unilateral bridge, side lying hip external rotation-clamshell, bridges on physio ball, bridge on physio ball with roll-in, bridge on physio ball alternating, hip hike. |  |
| Balance/proprioception:  Single leg standing balance (knee slightly flexed) static progressed to dynamic and level progressed to unsteady surface, Lateral step-overs, Joint position re-training | **Criteria to Progress:**  No swelling (Modified stroke test)  Flexion ROM within 10 Degree contra lateral side.  Extension ROM equal to contra lateral side. |
| Phase – III Late Post-operative phase (7-8 weeks) | |
| Range of motion/Mobility:  Rotational tibial mobilizations if limited ROM.  Cardio - Elliptical, stair climber, flutter kick swimming, pool jogging. | **Precautions:**  No active reactive swelling or joint pain that lasts more than 12 hours |
| Strengthening: Gym equipment: leg press machine, seated hamstring curl machine and hamstring curl machine, hip abductor and adductor machine, hip extension machine, roman chair, seated calf machine.  Progress intensity (strength) and duration (endurance) of exercises.  The following exercises to focus on proper control with emphasis on good proximal stability.  Squat to chair / Lateral lunges / Romanian deadlift / Single leg progression: partial weight bearing single leg press, slide board lunges, step ups and step ups with march, lateral step-ups, step downs, single leg squats, single leg wall slides / Knee Exercises for additional exercises and descriptions. | |
| Balance/proprioception:  Progress single limb balance including perturbation training | **Criteria to Progress:**  No swelling/ pain, Normal gait/ ROM equal to contra lateral side/ Normal joint position sense |
